# Detection and molecular analysis of betacoronaviruses (family *Coronaviridae*) in hedgehogs (*Erinaceus roumanicus*) in Hungary

**DOI:** 10.1007/s00705-025-06506-z

**Published:** 2026-01-07

**Authors:** Gábor Reuter, Chiara Ester Cora, Károly Takáts, Ákos Boros, Róbert Mátics, Benigna Balázs, Péter Pankovics

**Affiliations:** 1 Department of Medical Microbiology, Medical School, University of Pécs, Szigeti út 12, Pécs, H-7624 Hungary; 2 Hungarian Nature Research Society, Ajka, Hungary; 3 Department of Behavioural Sciences, Medical School, University of Pécs, Pécs, Hungary

## Abstract

In this study, the presence of a betacoronavirus (species *Betacoronavirus erinacei*, genus *Betacoronavirus*, family *Coronaviridae*) was investigated in faecal samples collected from northern white-breasted hedgehogs (*Erinaceus roumanicus*) in different sampling areas in Hungary using reverse transcription PCR (RT-PCR) and sequencing methods. A total of 23 (27.4%) of the 84 specimens were positive for betacoronaviruses (PX460030-PX460052), and the region of the viral genome encoding the complete spike protein was sequenced for eight of the positive samples. The complete spike genes from these eight specimens showed 91–94% nucleotide (nt) and 92–94% amino acid (aa) sequence identity to each other and 86–91% nt and 87–92% aa sequence identity to the other European hedgehog coronavirus strains. Hedgehog betacoronavirus is a common and widespread infectious agent in the three hedgehog species (*E. europaeus, E. amurensis*, and, based on this study, *E. roumanicus*) studied in Europe and Asia so far, with geographical and probably host-species-specific genetic lineages.

**Supplementary Information:**

The online version contains supplementary material available at 10.1007/s00705-025-06506-z.

At the beginning of the 21st century, three betacoronaviruses (SARS-CoV, MERS-CoV, and SARS-CoV-2; genus *Betacoronavirus*, family *Coronaviridae*) emerged pandemically in humans as a result of animal spillover [1], but our knowledge of the diversity and host spectrum of betacoronaviruses is still incomplete. In 2013, a novel betacoronavirus (hedgehog coronavirus 1) related to the human pathogen Middle East respiratory syndrome coronavirus (MERS-CoV) was discovered in a European hedgehog (*Erinaceus europaeus*) in Germany [2]. Since then, hedgehog betacoronaviruses (species *Betacoronavirus erinacei*) have also been detected in *Erinaceus europaeus* in the United Kingdom [3], Italy [4], Portugal [1], Poland [5, 6], and France [7], in *Erinaceus amurensis* in China [8, 9], and in an undetermined *Erinaceus* species in Russia [10]. The prevalence of hedgehog coronavirus is around 10.8% in the United Kingdom [3], 25% in Portugal [1] and Poland [5], and 60% in Germany and Italy [2, 11]. However, there have been no reports on the detection of betacoronaviruses in northern white-breasted hedgehogs (*Erinaceus roumanicus*) in Central Europe.

In this study, we report the detection, complete genome characterization, and molecular epidemiology of betacoronaviruses in northern white-breasted hedgehogs in Hungary.

Faecal samples (N = 84, ER1-ER84) were collected in different geographic regions of Hungary (Fig. 1, Supplementary Table S1) between 2023 and 2024 as described previously [12, 13]. Briefly, faecal specimens were collected from carcasses of road-killed hedgehogs or live animals that were being cared for in an official animal shelter and being prepared for release by qualified biologists with valid permission (the National Inspectorate for Environment, Nature and Water: 4018-4/2015). Faecal samples of 2–5 grams were transferred using disposable pipettes to sterile Eppendorf tubes, and DNA/RNAShield (Cambridge Bioscience) was added to prevent the degradation of nucleic acids. Viral RNA was isolated from these samples using TRI Reagent (MRC, Cincinnati, OH, USA), following the manufacturer’s instructions. The host origin (*Erinaceus roumanicus*) of the betacoronavirus-positive specimens was confirmed by PCR and Sanger sequencing, using the in-house-designed cytochrome-b-specific primers F (5’-GAGGCGCTACAGTCATTACTA-3’) and R (5’-CATTGACTTACAGGTCGGAAT) [13]. The samples were screened by RT-PCR using the generic screening primers betaCoV-screen-F (5’-TCAGTACCTGTTTCTGTMATTTATGAT-3’), corresponding to nt positions 23,536 − 23,562 and betaCoV-screen-R (5’-GCAGTRTACATAGCCTCCATATTAG-3’), corresponding to nt positions 24,398 − 24,374 of reference sequence 174/GER/2012 (KC545383). These primers were designed based on sequences of the spike protein coding regions of European hedgehog betacoronaviruses (N = 22) (Fig. 2) available in the GenBank database (accessed on June 2025) to yield an amplicon of 848–863 bp. The remaining part of the spike gene was determined for eight specimens using three additional RT-PCR and Sanger sequencing reactions using primers designed to generate overlapping PCR products (Supplementary Table S2). PCR products were sequenced directly in both directions using an automated DNA capillary sequencer (3500 Genetic Analyzer, Applied Biosystems, Japan). Next-generation sequencing (NGS) was performed for complete genome characterization, using total RNA isolated from a selected specimen (ER17) that was positive for betacoronavirus (ER17/2023/HUN, PX460032). The detailed description of the NGS and bioinformatics pipeline applied in this study can be found in our previous report [14]. Briefly, a 200-µl PBS-diluted sample (~ 40% V/V) was centrifuged at 6,000 × *g* for 5 min and passed through a 0.45-µm sterile polyethersulfone (PES) membrane filter (Millipore/Merck, Germany). The filtrate was incubated with a mixture of DNases and RNases at 37°C for 2 hours to digest free/unprotected nucleic acids. Enriched viral nucleic acids (both RNA and DNA) were then extracted using a Quick RNA Kit (Zymo Research, Irvine, CA, USA) according to the manufacturer’s instructions. An NGS library was constructed using an xGen DNA EZ Library Prep Kit (IDT, Coralville, IA, USA) and sequenced on a NovaSeq X Plus (Illumina, San Diego, CA, USA) platform. The resulting metagenomic reads were trimmed, assembled *de novo*, analysed, and classified using a previously described in-house bioinformatics pipeline [15] that includes adapter filtration and quality checking using FastQC v0.12.1, Spades assembler, Kaiju, and Diamond aligner software and makes use of the NCBI RefSeq databases.

**Fig. 1:**
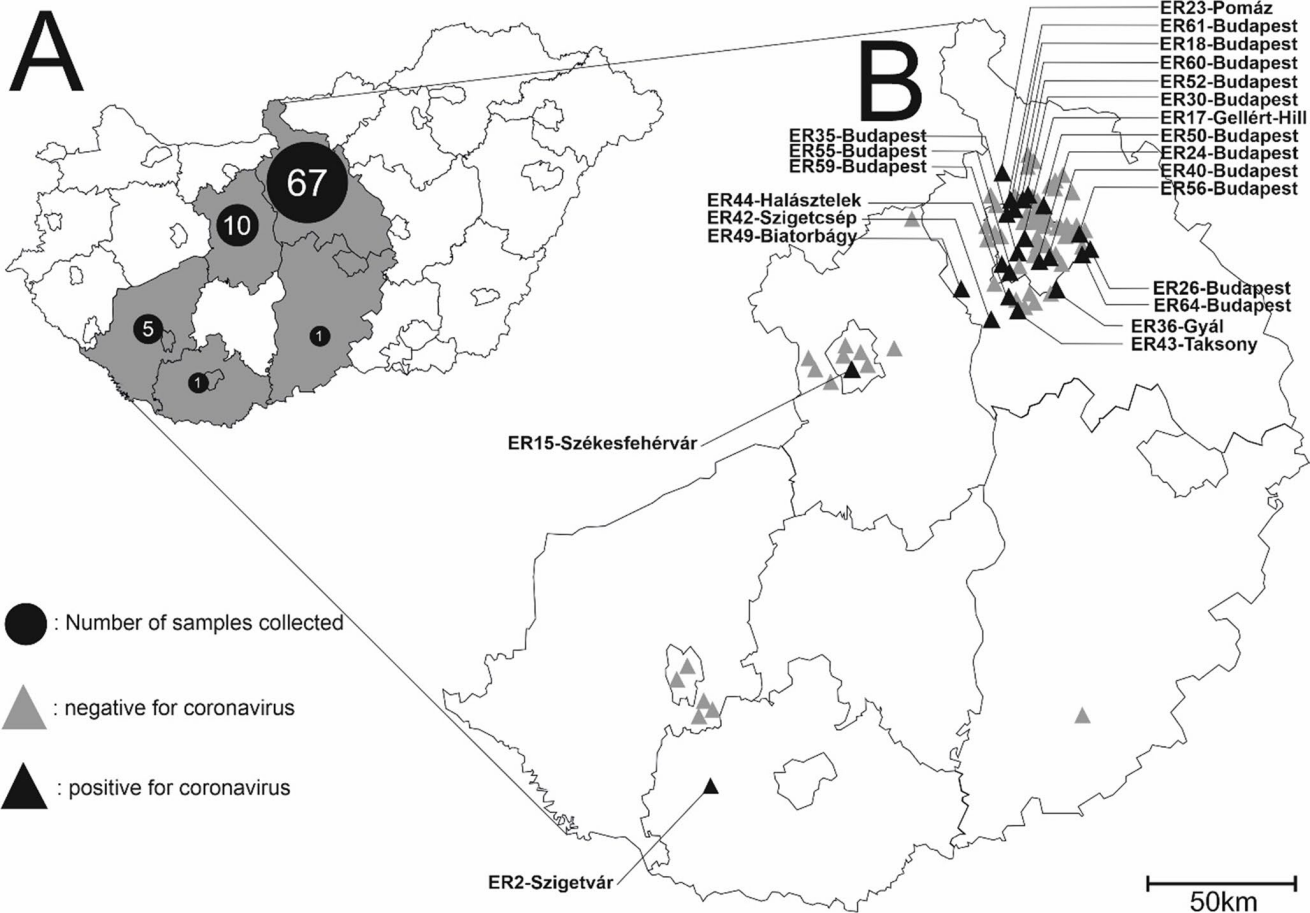
(**A**) Geographic distribution of the 84 sampled hedgehogs (*Erinaceus roumanicus*) in Hungary (gray area): Pest county and the capital Budapest, N = 67; Fejér County, N = 5; Baranya county, N = 1; Bács-Kiskun County, N = 1. (**B**) Specimens that tested negative (gray triangle, N = 61) and positive (black triangle, N = 23) by RT-PCR and Sanger sequencing for hedgehog betacoronavirus (species *Betacoronavirus erinacei*). The sample ID and the name of the place of sample collection are shown for positive specimens.

**Fig. 2:**
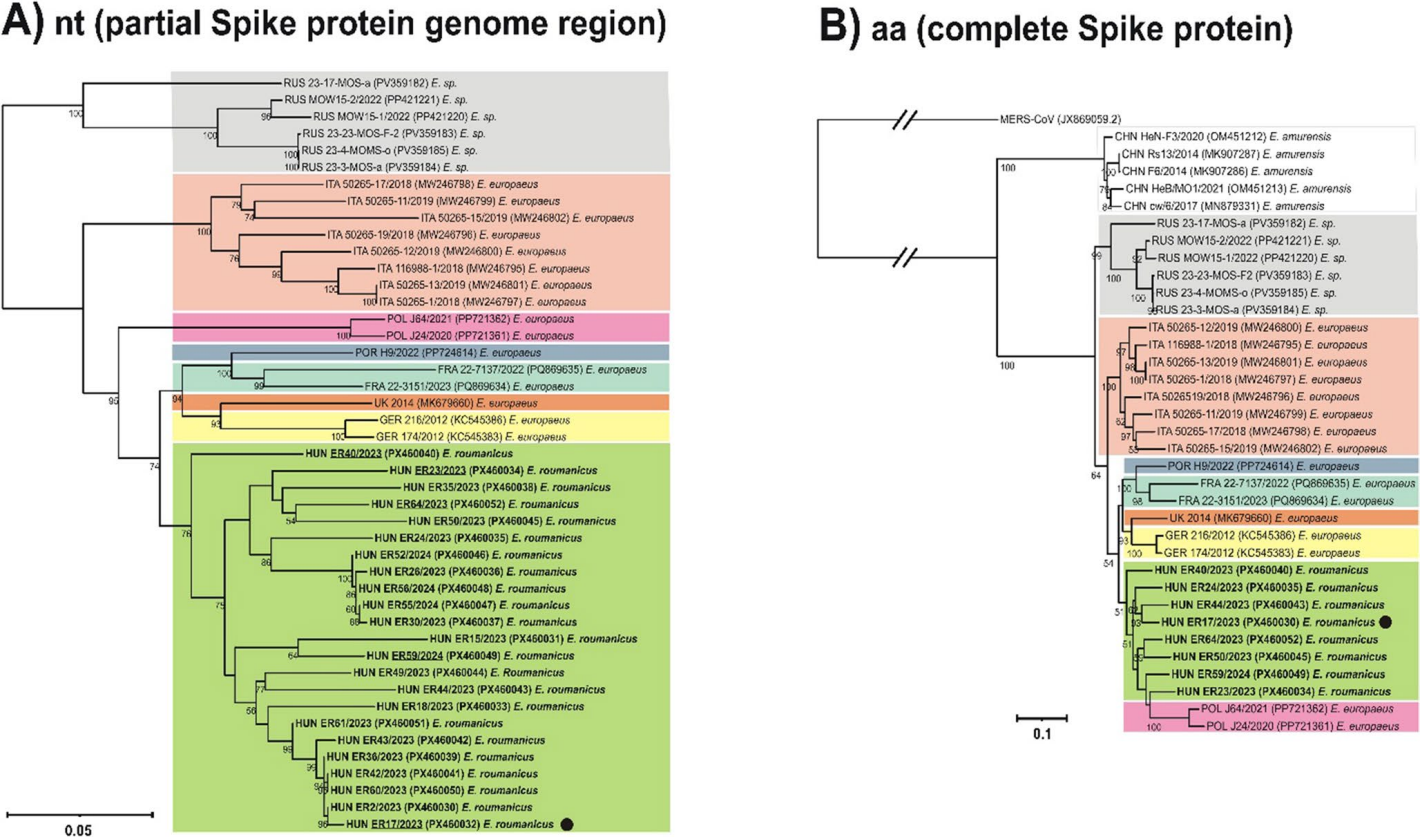
Phylogenetic analysis of hedgehog betacoronaviruses (subgenus *Merbecovirus*) from hedgehogs based on (**A**) a 793- to 814-nt portion of the region encoding the spike protein of European (N = 22) and the Hungarian (N = 23) hedgehog betacoronaviruses and (**B**) the complete amino acid sequences of the hedgehog betacoronavirus spike protein (N = 35) available in the GenBank database. The Hungarian strains selected for the determination of the sequence of the complete spike gene (~ 4,000 nucleotides) (N = 8, panel B) are underlined in panel A. Maximum-likelihood phylogenetic trees were generated from ClustalX alignments of the nucleotide (left) and amino acid (right) sequences, using the best-fit models selected by IQ-Tree [16]. Maximum-likelihood trees with 1,000 ultrafast bootstrap replicates were produced using the IQ-Tree web server [17] and visualized using iTOL and CorelDRAW Standard 2020. The strains are named as follows: country of origin (CHN, China; FRA, France; GER, Germany; HUN, Hungary; ITA, Italy; POL, Poland; POR, Portugal; RUS, Russia; UK, United Kingdom), strain name in GenBank, collection year if available, GenBank accession number, and original hedgehog host species. The countries of origin are indicated by different background colors. Bold letters indicate the study sequences from Hungary. The black dot (●) indicates the complete betaretrovirus genome sequence (ER17/HUN/2023) from Hungary. E. = *Erinaceus*, sp. = species

A total of 23 (27.4%) of the 84 faecal specimens were RT-PCR-positive for betacoronaviruses using the screening primers designed for the spike protein coding region (Supplementary Table S1). The sequences of all 23 PCR products were determined by the Sanger method, and for eight of the betacoronavirus-positive specimens (ER17, ER23, ER24, ER40, ER44, ER50, ER59, and ER64), the complete (~ 4,000-nt-long) region encoding the spike protein was sequenced. In addition, the nearly complete 30,150-nt-long RNA genome sequence of strain ER17 (PX460032) was determined. Hedgehog-betacoronavirus-positive specimens were found in three geographic regions (in three of the five counties tested), with predominance in the districts including the capital Budapest and the surrounding area, but the number of samples collected varied greatly (Fig. 1).

An alignment of a 802- to 814-nucleotide-long portion of the region encoding the spike protein showed that there was 90–99% nt and 89–100% aa sequence identity among the 23 Hungarian strains and 84–93% nt and 84–95% aa sequence identity between the Hungarian strains and the 22 hedgehog betacoronavirus 1 strains from Europe (sequences obtained from the GenBank database in August 2025). Phylogenetic analysis based on this alignment showed that the 23 Hungarian study strains formed a common phylogenetic lineage (Fig. 2A). Interestingly, the hedgehog coronavirus sequences were generally well separated by geographic origin (by country) (Fig. 2A).

Comparisons of the approximately 4,000-nucleotide-long complete spike protein coding region showed 91–94% nt and 92–94% aa sequence identity among the eight selected Hungarian strains from *Erinaceus roumanicus*, and 86–91% nt and 87–92% aa sequence identity between the Hungarian strains and 22 other European hedgehog coronavirus strains from *Erinaceus europaeus*. The aa sequence identity in the spike protein was 76–78% between the eight Hungarian strains and five Chinese hedgehog coronavirus strains from *Erinaceus amurensis*. In addition to amino acid substitutions, unique nucleotide insertions were detected in the Hungarian coronavirus strains, including a 9-nucleotide-long insertion (TCGAGTTTG, encoding the amino acids SSL) in ER64/HUN/2024 (PX460052) at nt positions 2,065 − 2,073 in the spike protein coding region. Phylogenetic analysis based on complete amino acid sequences of the spike protein showed a separation of the coronavirus strains by country and probably also by hedgehog species (Fig. 2B).

Using GenBank BLASTn (accessed on Sept 2025), ER17/HUN/2023 (PX460032) was found to have the most nucleotide sequence similarity (93.6% identity) to EriCoV/2012 − 216/GER/2012 (KC545386) from Germany [2] based on the complete hedgehog coronavirus genome sequence. The amino acid sequences of the ORF1ab, ORF1a, spike, ORF3a, ORF3b, ORF4a, ORF4b, ORF5, envelope, membrane, nucleocapsid, and ORF8b proteins of ER17/HUN/2023 (PX460032) and EriCov/2012 − 216/GER/2012 (KC545386) were found to be 95%, 94%, 91%, 91%, 81%, 83%, 82%, 87%, 93%, 96%, 95%, and 83% identical, respectively. The insertion of a 411- to 630-bp-long sequence orthologous to the host CD200 gene, located between the spike and ORF3a genes, which was previously reported in EriCoV/Italy/50265-19/2018 (MW246796) and seven other strains sampled in northern Italy [18], was not observed in ER17/HUN/2023.

Coinfection with hedgehog coronavirus 1 and a mammarenavirus (family *Arenaviridae*) [13] was detected in one faecal specimen (ER15) (Supplementary Table S1).

In this study, the prevalence and genetic diversity of hedgehog coronavirus were investigated in a previously uninvestigated hedgehog species, *Erinaceus roumanicus. E. roumanicus* is the only hedgehog species native to Hungary. Hedgehog coronavirus was detected by RT-PCR and Sanger sequencing in a high percentage (27.4%) of hedgehog faecal samples collected over a wide geographic area. This high prevalence rate is in line with previous reports in *E. europaeus* [2, 3, 11]. This study increases the number of known hedgehog coronavirus sequences by 23 partial spike protein sequences, eight complete spike protein sequences and one complete genome sequence.

This new information about the sequence diversity of the coronavirus spike protein can potentially contribute to the design of more-specific screening primers as well as the study of viral replication, receptor interaction, and viral evolution. Coronaviruses use the viral spike glycoprotein to bind the host cell receptor via its receptor-binding domain (RBD). The hedgehog coronavirus receptor was discovered very recently [19]. The hedgehog aminopeptidase N (APN) receptor serves as a primary receptor, and *in vitro* infection occurs more efficiently at temperatures lower than 37°C, which has been observed in other hibernating animals [19]. Previously, the receptor specificity of the hedgehog coronavirus spike protein was found to be unexpectedly narrow, with only APN from the Cape elephant shrew (*Elephantulus edwardii*), brown rat (*Rattus norvegicus*), and domestic cat (*Felis catus*), in addition to hedgehog APN, supporting viral entry [19]. While hedgehog coronavirus is closely related phylogenetically to highly pathogenic MERS-CoV in humans, the hedgehog coronavirus RBD and APN receptor complex represent a potential species barrier at the receptor-binding level, which would likely prevent the hedgehog coronavirus from spilling over into humans [19]. However, the enormous quasispecies diversity and recombination potential of betacoronaviruses could change this understanding.

So far, hedgehog coronavirus has been reported in nine countries (eight of which are in Europe). Based on phylogenetic analysis of the spike protein region and deduced protein sequences, genetic lineages of hedgehog coronavirus can be separated by geographical region (country). Separation by host species is also observed in the case of *E. europaeus* (Europe) and *E. amurensis* (Asia), but it is not pronounced in the case of the European hedgehog coronaviruses from the hedgehog species *E. europaeus* and *E. roumanicus* studied so far.

Surprisingly high prevalence, prolonged and intermittent virus shedding [4], and genetic diversity of hedgehog coronaviruses were observed in studies at certain sampling times. This raises the possibility of persistent infection and viral shedding in addition to acute infection in hedgehogs. The epidemiology, pathogenesis, seroepidemiology, and reason for the high prevalence of hedgehog coronavirus in hedgehogs are unknown and require further investigation.

## Electronic Supplementary Material

Below is the link to the electronic supplementary material

Supplementary Material 1

Supplementary Material 2

## Data Availability

The nucleotide sequence data reported here are available in the DDBJ/EMBL/GenBank databases under the accession numbers PX460030-PX460052.
