# Supplementary material for: Detection and molecular analysis of betacoronaviruses (family Coronaviridae) in hedgehogs (Erinaceus roumanicus) in Hungary
Source: Arch Virol. 2026 Jan 7;171(2):44. doi: 10.1007/s00705-025-06506-z (PMC12779734; doi:10.1007/s00705-025-06506-z)
Supplement: Supplementary file 1 — Supplementary Material 1 [file 705_2025_6506_MOESM1_ESM.docx]

**Table S1.** Summary of the specimens from hedgehogs (*Erinaceus roumanicus*) and the RT-PCR results for hedgehog betacoronavirus 1.

* indicates betacoronavirus co-infection with mammarenavirus (strain ER15/2023/HUN, PQ441961-PQ441962) [13].

| Sample ID | Sample type | Sampling time | Geographical location | RT-PCR screening results |
| --- | --- | --- | --- | --- |
| ER1 | stool | Jan 15, 2023 | Csór  47°12′14″N 18°15′25″E | negative |
| ER2 | stool | Jan 15, 2023 | Szigetvár  46°02′51″N 17°47′58″E | **positive** |
| ER3 | stool | Jan 17, 2023 | Pákozd  47°13′16″N 18°32′42″E | negative |
| ER4 | stool | Apr 1, 2023 | Simonfa  46°17′01″N 17°49′23″E | negative |
| ER5 | stool | Apr 1, 2023 | Simonfa  46°17′01″N 17°49′23″E | negative |
| ER6 | stool | May 21, 2023 | Érd  47°23′N.18°55′E | negative |
| ER7 | stool | June 6, 2023 | Veresegyháza  47°39′25″N 19°17′05″E | negative |
| ER8 | stool | June 8, 2023 | Úrhida  47°07′50″N 18°19′55″E | negative |
| ER9 | stool | June 21, 2023 | Csór  47°12′14″N 18°15′25″E | negative |
| ER10 | stool | June 21, 2023 | Székesfehérvár  47°11′20″N 18°24′50″E | negative |
| ER11 | stool | June 25, 2023 | 16th district of Budapest  47°31′13″N 19°10′26″E | negative |
| ER12 | stool | June 25, 2023 | Székesfehérvár  47°11′20″N 18°24′50″E | negative |
| ER13 | stool | June 26, 2023 | 4th district of Budapest  47° 34′N 19°05′E | negative |
| ER14 | stool | June 27, 2023 | Simonfa  46°17′01″N 17°49′23″E | negative |
| ER15 | stool | June 27, 2023 | Székesfehérvár  47°11′20″N 18°24′50″E | **positive*** |
| ER16 | stool | June 28, 2023 | Székesfehérvár  47°11′20″N 18°24′50″E | negative |
| ER17 | stool | July 1, 2023 | Gellért Hill Budapest  47°29′13″N 19°02′50″E | **positive** |
| ER18 | stool | July 1, 2023 | 3rd district of Budapest  47°34′N 19°02′E | **positive** |
| ER19 | stool | July 1, 2023 | 4th district of Budapest  47° 34′N 19°05′E | negative |
| ER20 | stool | July 1, 2023 | Székesfehérvár  47°11′20″N 18°24′50″E | negative |
| ER21 | stool | July 2, 2023 | 16th district of Budapest  47°31′13″N 19°10′26″E | negative |
| ER22 | stool | July 3, 2023 | 12th district of Budapest  47°30′N. 19° 00′E | negative |
| ER23 | stool | July 28, 2023 | Pomáz  47°38′51″N 19°01′37″E | **positive** |
| ER24 | stool | Aug 8, 2023 | 21st district of Budapest  47°25′N 19°05′E | **positive** |
| ER25 | stool | Aug 9, 2023 | 16th district of Budapest  47°31′13″N 19°10′26″E | negative |
| ER26 | stool | Aug 9, 2023 | 17th district of Budapest  47°28′49″N 19°16'00″E | **positive** |
| ER27 | stool | Aug 13, 2023 | Budapest  47°29′54″N 19°02′27″E | negative |
| ER28 | stool | Aug 15, 2023 | Budapest 47°29′54″N19°02′27″E | negative |
| ER29 | stool | Aug 16, 2023 | 3rd district of Budapest  47°34′N 19°02′E | negative |
| ER30 | stool | Aug 19, 2023 | 4th district of Budapest  47° 34′N 19°05′E | **positive** |
| ER31 | stool | Aug 30, 2023 | 16th district of Budapest  47°31′13″N 19°10′26″E | negative |
| ER32 | stool | Aug 31, 2023 | 14th district of Budapest  47°30′45″N 19°06′30″E | negative |
| ER33 | stool | Aug 31, 2023 | Fót  47°36′33″N 19°11′34″E | negative |
| ER34 | stool | Aug 31, 2023 | 15th district of Budapest  47°28′N 19°05′E | negative |
| ER35 | stool | Sept 3, 2023 | 11th district of Budapest 47°28′30″N 19°02′24″E | **positive** |
| ER36 | stool | Sept 4, 2023 | Gyál  47°22′56″N 19°12′49″E | **positive** |
| ER37 | stool | Sept 5, 2023 | 18th district of Budapest  47°26′38″N 19°10′35″E | negative |
| ER38 | stool | Sept 8, 2023 | 2nd district of Budapest  47°31′N 19°01′E | negative |
| ER39 | stool | Sept 15, 2023 | Szár  47°28′39″N 18°30′58″E | negative |
| ER40 | stool | Sept 27, 2023 | 20th district of Budapest  47°26′06″N 19°07′00″E | **positive** |
| ER41 | stool | Oct 2, 2023 | Fót  47°36′33″N 19°11′34″E | negative |
| ER42 | stool | Oct 7, 2023 | Szigetcsép  47°16′N 18°59′E | **positive** |
| ER43 | stool | Oct 15, 2023 | Taksony  47°19′55″N 19°03′47″E | **positive** |
| ER44 | stool | Oct 19, 2023 | Halásztelek  47°21′39″N 18°59′16″E | **positive** |
| ER45 | stool | Oct 23, 2023 | 16th district of Budapest  47°31′13″N 19°10′26″E | negative |
| ER46 | stool | Oct 24, 2023 | Budapest  47°29′54″N 19°02′27″E | negative |
| ER47 | stool | Nov 11, 2023 | 16th district of Budapest  47°31′13″N 19°10′26″E | negative |
| ER48 | stool | Nov 13, 2023 | Dunaharaszti  47°21′19″N 19°05′04″E | negative |
| ER49 | stool | Nov 22, 2023 | Biatorbágy  47°28′27″N 18°49′25″E | **positive** |
| ER50 | stool | Dec 21, 2023 | 15th district of Budapest  47°33′32″N 19°07′10″E | **positive** |
| ER51 | stool | Jan 4, 2024 | Budapest  47°29′54″N19°02′27″E | negative |
| ER52 | stool | Jan 7, 2024 | 4th district of Budapest  47° 34′N 19°05′E | **positive** |
| ER53 | stool | Jan 22, 2024 | Biatorbágy  47°28′27″N 18°49′25″E | negative |
| ER54 | stool | Jan 22, 2024 | Szigetszentmiklós  47°20′43″N 19°02′54″E | negative |
| ER55 | stool | Jan 22, 2024 | Budapest  47°29′54″N 19°02′27″E | **positive** |
| ER56 | stool | Jan 24, 2024 | 16th district of Budapest  47°31′13″N 19°10′26″E | **positive** |
| ER57 | stool | Jan 27, 2024 | 12th district of Budapest  47°30′N. 19° 00′E | negative |
| ER58 | stool | Febr 2, 2024 | 12th district of Budapest  47°30′N 19° 00′E | negative |
| ER59 | stool | Febr 8, 2024 | Budapest  47°29′54″N 19°02′27″E | **positive** |
| ER60 | stool | Jul 6, 2023 | 3rd district of Budapest  47°34′N 19°02′E | **positive** |
| ER61 | stool | Jul 6, 2023 | 3rd district of Budapest  47°34′N 19°02′E | **positive** |
| ER62 | stool | Sept 9, 2023 | Göd  47°41′26″N 19°08′04″E | negative |
| ER63 | stool | Sept 18, 2023 | Kaposvár  46°22′N 17°47′E | negative |
| ER64 | stool | Sept 23, 2023 | 17th district of Budapest  47°28′49″N 19°16'00″E | **positive** |
| ER65 | stool | Sept 23, 2023 | Dunaharaszti  47°21′19″N 19°05′04″E | negative |
| ER66 | stool | Sept 23, 2023 | 19th district of Budapest  47°26′59″N 19°07′59″E | negative |
| ER67 | stool | Oct 05, 2023 | 2nd district of Budapest  47°31′N 19°01′E | negative |
| ER68 | stool | Oct 26, 2023 | Fót  47°36′33″N 19°11′34″E | negative |
| ER69 | stool | Dec 14, 2023 | Budafok  47°25′12″N 19°01′42″E | negative |
| ER70 | stool | Feb 15, 2024 | 17th district of Budapest  47°28′49″N 19°16'00″E | negative |
| ER71 | stool | Feb 15, 2024 | Dunaharaszti  47°21′19″N 19°05′04″E | negative |
| ER72 | stool | Mar 15, 2024 | 15th district of Budapest  47°33′32″N 19°07′10″E | negative |
| ER73 | stool | Mar 24, 2024 | Göd  47°41′26″N 19°08′04″E | negative |
| ER74 | stool | Apr 10, 2024 | 16th district of Budapest  47°31′13″N 19°10′26″E | negative |
| ER75 | stool | Apr 10, 2024 | 15th district of Budapest  47°33′32″N 19°07′10″E | negative |
| ER76 | stool | Apr 10, 2024 | 15th district of Budapest  47°33′32″N 19°07′10″E | negative |
| ER77 | stool | May 06, 2024 | 15th district of Budapest  47°33′32″N 19°07′10″E | negative |
| ER78 | stool | May 16, 2024 | 20th district of Budapest  47°26′06″N 19°07′00″E | negative |
| ER79 | stool | May 20, 2024 | Kiskunhalas  46°25′55″N 19°29′18″E | negative |
| ER80 | stool | May 21, 2024 | Göd  47°41′26″N 19°08′04″E | negative |
| ER81 | stool | May 21, 2024 | Fót  47°36′33″N 19°11′34″E | negative |
| ER82 | stool | May 21, 2024 | 15th district of Budapest  47°33′32″N 19°07′10″E | negative |
| ER83 | stool | May 21, 2024 | Gyál  47°22′56″N 19°12′49″E | negative |
| ER84 | stool | May 21, 2024 | Kaposvár  46°22′N 17°47′E | negative |
