# Supplementary material for: Detection and molecular analysis of betacoronaviruses (family Coronaviridae) in hedgehogs (Erinaceus roumanicus) in Hungary
Source: Arch Virol. 2026 Jan 7;171(2):44. doi: 10.1007/s00705-025-06506-z (PMC12779734; doi:10.1007/s00705-025-06506-z)
Supplement: Supplementary file 2 — Supplementary Material 2 [file 705_2025_6506_MOESM2_ESM.docx]

**Table S2.** Primer pairs designed for the detection (“screen”) and determination (“spike”) of the complete Spike protein genome region of hedgehog betacoronaviruses.

| primer name | primer sequences (5’-3’) | nt positions in reference strain KC545383 | PCR product size (bp) |
| --- | --- | --- | --- |
| betaCoV-screen-R | 5’-GCAGTRTACATAGCCTCCATATTAG-3’ | 24,374-24,398 | 848-863 |
| betaCoV-screen-F | 5’-TCAGTACCTGTTTCTGTMATTTATGAT-3’ | 23,536-23,562 |  |
| betaCoV-spike1-R | 5’-AACAATACATCTAATTCACATTCT-3’ | 22,716-22,739 | 1,137-1,152 |
| betaCoV-spike1-F | 5’-TTRCTYATACGTGACAATGATAC-3’ | 21,594-21,616 |  |
| betaCoV-spike2-R | 5’-CAATAACACAACCAACAGCAGTT-3’ | 23,688-23,710 | 1,088-1,106 |
| betaCoV-spike2-F | 5’-ATGCTCTTATGGBCAATTTGATAT-3’ | 22,617-22,640 |  |
| betaCoV-spike3-R | 5’-TCATCATACTTRTCACAACAACG-3’ | 25,546-25,568 | 1,268-1,271 |
| betaCoV-spike3-F | 5’-GGACCACCTAGTGCTAGGGA-3’ | 24,298-24,317 |  |
